# Towards long-read metagenomics: complete assembly of three novel genomes from bacteria dependent on a diazotrophic cyanobacterium in a freshwater lake co-culture

**DOI:** 10.1186/s40793-017-0224-8

**Published:** 2017-01-19

**Authors:** Connor B. Driscoll, Timothy G. Otten, Nathan M. Brown, Theo W. Dreher

**Affiliations:** 1 Department of Microbiology, Oregon State University, 226 Nash Hall, Corvallis, OR 97331 USA; 2 Center for Genome Research and Biocomputing, Oregon State University, Corvallis, OR 97331 USA

**Keywords:** Aerobic anoxygenic phototroph, *Aphanizomenon flos-aquae*, *Hyphomonadaceae*, *Betaproteobacterium*, *Bacteroidetes*, PacBio SMRT sequencing

## Abstract

Here we report three complete bacterial genome assemblies from a PacBio shotgun metagenome of a co-culture from Upper Klamath Lake, OR. Genome annotations and culture conditions indicate these bacteria are dependent on carbon and nitrogen fixation from the cyanobacterium *Aphanizomenon flos-aquae,* whose genome was assembled to draft-quality*.* Due to their taxonomic novelty relative to previously sequenced bacteria, we have temporarily designated these bacteria as *incertae sedis Hyphomonadaceae* strain UKL13-1 (3,501,508 bp and 56.12% GC), *incertae sedis* Betaproteobacterium strain UKL13-2 (3,387,087 bp and 54.98% GC), and *incertae sedis Bacteroidetes* strain UKL13-3 (3,236,529 bp and 37.33% GC). Each genome consists of a single circular chromosome with no identified plasmids. When compared with binned Illumina assemblies of the same three genomes, there was ~7% discrepancy in total genome length. Gaps where Illumina assemblies broke were often due to repetitive elements. Within these missing sequences were essential genes and genes associated with a variety of functional categories. Annotated gene content reveals that both *Proteobacteria* are aerobic anoxygenic phototrophs, with Betaproteobacterium UKL13-2 potentially capable of phototrophic oxidation of sulfur compounds. Both proteobacterial genomes contain transporters suggesting they are scavenging fixed nitrogen from *A. flos-aquae* in the form of ammonium. *Bacteroidetes* UKL13-3 has few completely annotated biosynthetic pathways, and has a comparatively higher proportion of unannotated genes. The genomes were detected in only a few other freshwater metagenomes, suggesting that these bacteria are not ubiquitous in freshwater systems. Our results indicate that long-read sequencing is a viable method for sequencing dominant members from low-diversity microbial communities, and should be considered for environmental metagenomics when conditions meet these requirements.

**Electronic supplementary material:**

The online version of this article (doi:10.1186/s40793-017-0224-8) contains supplementary material, which is available to authorized users.

## Introduction

Metagenomic sequencing is the process of sampling DNA sequences from multiple genomes in a community of organisms, and has been applied to many environmental samples to assess both functional diversity and species richness of microbial communities [1, 2]. Recently, there has been a progression in metagenomic approaches associated with advances in sequencing technologies. Next-generation sequencing methods [3] such as 454 and Illumina HiSeq/MiSeq have greatly reduced sequencing costs per base relative to Sanger sequencing due to increased throughput, which has facilitated shotgun metagenomics (randomly sequencing all DNA in a sample). This has provided several advantages over amplicon sequencing. For example, focus has shifted from assigning taxa using single genes to using multiple genes and sequence composition instead [2, 4]. It has also permitted functional characterization of individual representatives or whole microbial communities [5, 6].

However, there are technical hurdles associated with short-read sequencing. Specifically, assembling short reads (50-300 bp) into contiguous sequences (contigs) rarely leads to complete genome assemblies due to repetitive genomic elements such as 16S rRNA genes and IS elements [7] that are 1 kb or greater in length. There are two consequences as a result. First, closing draft genomes by primer walking requires considerable manual effort and time. Second, if closure is not possible, contigs must be clustered and binned using methods like differential coverage [8], co-abundance [5, 9, 10], or gene/nucleotide composition [11]. While useful, these methods are often not comprehensive and become even more difficult to implement when used in a metagenomic context, where multiple genomes (sometimes from closely related organisms) must be delineated [12].

Single-molecule real time sequencing technologies, such as PacBio and Oxford Nanopore, are part of the third-generation sequencing wave [7]. These sequencers produce average read lengths in the 5–50 kb range, with ~50% of reads longer than 14 kb [13], which exceeds the size of repetitive elements in the average bacterial genome. Although more error-prone, these longer reads have proven advantageous for assembling closed genomes if sequencing depth is high enough to allow error correction [14]. To date, long-read sequencing has rarely been used for metagenomics for several reasons: 1) the amount of sequence data returned is a fraction of an Illumina run (up to 750 Gb/flow cell of Illumina HiSeq 3000 vs. up to 1 Gb/SMRT cell of PacBio Sequel based on company specifications), 2) the sequencing cost per base pair is higher, and 3) PacBio does not rely upon DNA amplification, so high concentrations of raw DNA are required. Due to these limitations, long-read metagenomics has so far been limited to whole-16S amplicon sequencing [15] and to improving binning from fragmented (short-read) assemblies [16].

Here, we have generated a PacBio shotgun metagenome from a non-axenic cyanobacterium culture established in summer 2013 originating from Upper Klamath Lake, OR. In this freshwater lake, the N_2_-fixing filamentous cyanobacterium *Aphanizomenon* *flos-aquae* blooms annually. These blooms are harvested and sold as nutritional supplements. Little is known about the co-occurring microbial community in this lake, whose composition could be influenced by the presence of *A. flos-aquae* as the dominant primary producer [17, 18]. By applying a selective growth medium lacking nitrogen, our goal was to sequence and assemble complete genomes from a relatively simple community, in turn assessing the possibility for using PacBio shotgun sequencing for environmental metagenomics. We closed three novel bacterial genomes, which provide insight into putative metabolic dependencies of these bacteria on *A. flos-aquae* in the co-culture*.* However, we were unable to close the *A. flos-aquae* genome, which is in draft quality and will be discussed elsewhere.

## Organism information

### Classification and features

**Fig. 1:**
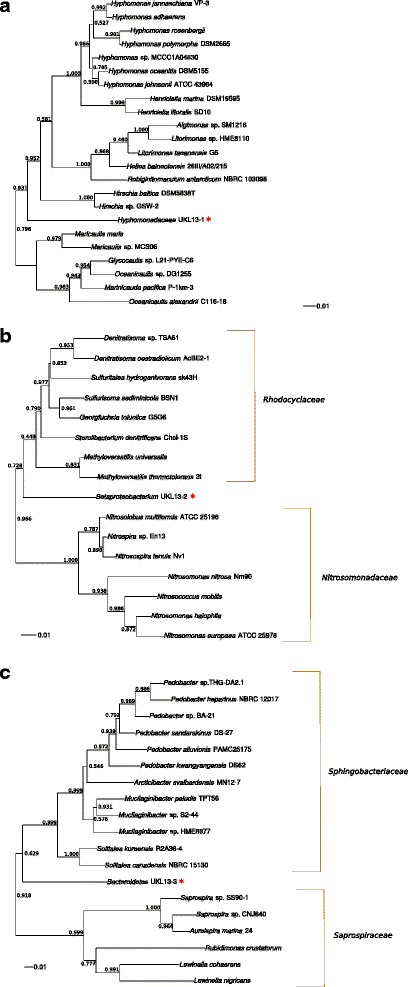
**a** *Hyphomonadaceae* UKL13-1 1 16S phylogenetic tree. The novel genome is placed between two branches of the *Hyphomonadaceae*, current members of which are almost all marine bacteria [60]. They are strict aerobes inhabiting oligotrophic niches, often reduce nitrate, and are flagellated, though some produce stalks to become sessile. None are known to be photosynthetic [60]. The scale represents substitutions per nucleotide. **b** *Betaproteobacterium* UKL13-2 16S phylogenetic tree. The novel genome is placed between two betaproteobacterial families, and is not part of the widely distributed bet or Pnec clades found in freshwater lakes across the world (Additional file 1: Table S1) [43]. The *Nitrosomonadaceae* are ammonia oxidizers [61], while the *Rhodocyclaceae* include chemolithotrophs and methylotrophs [62]. None are known to be photosynthetic. The *amo*A gene common to ammonia oxidizers was not detected in the *Betaproteobacterium* UKL13-2 genome. **c** *Bacteroidetes* UKL13-3 16S phylogenetic tree. The novel genome is placed between two *Bacteroidetes* families

The taxonomic placement of each genome was assessed three ways (Additional file 1: Table S1). We used the SILVA SSU Ref NR database (accessed on March 9, 2016) to search for significant 16S rDNA matches [19]. Also, we generated 16S phylogenetic trees for each genome, using the SINA aligner [20] and FastTree [21], with all classified *Alphaproteobacteria* *,* *Betaproteobacteria* *,* and *Bacteroidetes* representatives in SILVA, shown with their nearest groups in Fig. 1a-c. For the second taxonomic placement method, we used PhyloPythiaS+ [4], which searches for genomes with similar k-mer composition. The third method, Phylosift [22], is a pipeline that aligns 40 marker genes to generate a weighted probability score for specific taxonomic assignments. Due to lack of similarity with previously-classified bacterial representatives, these approaches were unable to assign these genomes to genus or species levels. Phylogenetic analysis of 16S rRNA genes placed each of the novel bacteria between established clades within or between families, with 0.11–0.13 average substitutions per site to the nearest neighbor's 16S gene (Fig. 1a-c). Phylogenomic analyses (PhylopythiaS+ and Phylosift) were also unable to find close relatives, resulting in the genomes being placed at higher taxonomic levels than genus (Additional file 1: Table S1). We have therefore used this information to designate these organisms as *incertae sedis* (of uncertain placement). While *Candidatus* designations are often assigned to unplaced taxa, the International Code of Nomenclature for Prokaryotes requires the *Candidatus* usage to be accompanied by phenotypic information [23], which we did not have available. We assigned the temporary strain names *Hyphomonadaceae* UKL13-1, Betaproteobacterium UKL13-2, and *Bacteroidetes* UKL13-3 until further representative sequences become available to guide the naming of new genera as appropriate. Minimum Information about the Genome Sequences is summarized in Table 1.

**Table 1:** Classification and general features of UKL genomes according to MIGS specifications [63]

MIGS ID	Property	*Hyphomonadaceae UKL13-1*	Evidence code^a^	*Betaproteobacterium UKL13-2*	Evidence code	*Bacteroidetes UKL13-3*	Evidence code^a^
	Classification	Domain *Bacteria*	TAS [64]	Domain *Bacteria*	TAS [64]	Domain *Bacteria*	TAS [64]
		Phylum *Proteobacteria*	TAS [65]	Phylum *Proteobacteria*	TAS [65]	Phylum *Bacteroidetes*	TAS [66]
		Class *Alphaproteobacteria*	TAS [67]	Class *Betaproteobacteria*	TAS [68]	Class *incertae sedis*	NAS
		Order *Rhodobacterales*	TAS [69]	Order *incertae sedis*	NAS	Order *incertae sedis*	NAS
		Family *Hyphomonadaceae*	TAS [70]	Family *incertae sedis*	NAS	Family *incertae sedis*	NAS
		Genus *incertae sedis*	NAS	Genus *incertae sedis*	NAS	Genus *incertae sedis*	NAS
		Species *incertae sedis*	NAS	Species *incertae sedis*	NAS	Species *incertae sedis*	NAS
		Strain UKL13-1	NAS	Strain UKL13-2	NAS	Strain UKL13-3	NAS
	Gram stain	Unknown	NAS	Unknown	NAS	Unknown	NAS
	Cell shape	Unknown	NAS	Unknown	NAS	Unknown	NAS
	Motility	Unknown	NAS	Unknown	NAS	Unknown	NAS
	Sporulation	Unknown	NAS	Unknown	NAS	Unknown	NAS
	Temperature range	22-28 °C	NAS	22-28 °C	NAS	22-28 °C	NAS
	Optimum temperature	Unknown	NAS	Unknown	NAS	Unknown	NAS
	pH range; Optimum	7.5-8.5; Unknown	NAS	7.5-8.5; Unknown	NAS	7.5-8.5; Unknown	NAS
	Carbon source	Unknown	NAS	Unknown	NAS	Unknown	NAS
	Terminal electron acceptor	Unknown	NAS	Unknown	NAS	Unknown	NAS
MIGS-6	Habitat	Freshwater lake	NAS	Freshwater lake	NAS	Freshwater lake	NAS
MIGS-6.3	Salinity	0.25%	NAS	0.25%	NAS	0.25%	NAS
MIGS-22	Oxygen requirement	Aerobic	NAS	Aerobic	NAS	Aerobic	NAS
MIGS-15	Biotic relationship	Syntrophic	TAS [71]	Syntrophic	TAS [71]	Syntrophic	TAS [71]
MIGS-14	Pathogenicity	Unknown	NAS	Unknown	NAS	Unknown	NAS
MIGS-4	Geographic location	Upper Klamath Lake, Oregon, USA	NAS	Upper Klamath Lake, Oregon, USA	NAS	Upper Klamath Lake, Oregon, USA	NAS
MIGS-5	Sample collection	Aug 6, 2013	NAS	Aug 6, 2013	NAS	Aug 6, 2013	NAS
MIGS-4.1	Latitude	42°22' N	NAS	42°22' N	NAS	42°22' N	NAS
MIGS-4.2	Longitude	-121°55' W	NAS	-121°55' W	NAS	-121°55' W	NAS
MIGS-4.4	Altitude	1,260 m	NAS	1,260 m	NAS	1,260 m	NAS

^a^Evidence Codes *IDA* Inferred from Direct Assay, *TAS* Traceable Author Statement (i.e., a direct report exists in the literature), *NAS* Non-traceable Author Statement (i.e., not directly observed for the living, isolated sample, but based on a generally accepted property for the species, or anecdotal evidence). These evidence codes are derived from the Gene Ontology project

Although we initiated and maintained this mixed-community culture for 1 year, the culture was lost  and we did not obtain physiological information regarding these organisms. Sustaining long-term *A. flos-aquae* cultures is often difficult, and it is common for cultures to die. In the absence of phenotypic information, we discuss insights from the genome annotations for the three novel bacteria.

## Genome sequencing information

### Genome project history

Cultures were initiated from UKL, where annual *A. flos-aquae* blooms constitute a serious ecological disturbance but are also harvested and sold as nutritional supplements. The genome sequences were deposited to DDBJ/EMBL/GenBank under the accessions CP012156, CP012157, and CP012155 for the *Hyphomonadaceae* UKL13-1, Betaproteobacterium UKL13-2, and *Bacteroidetes* UKL13-3 genomes, respectively. Project information is summarized in Table 2.

**Table 2:** Project information

MIGS ID	Property	*Hyphomonadaceae* UKL13-1	*Betaproteobacterium* UKL13-2	*Bacteroidetes* UKL13-3
MIGS-31	Finishing quality	Complete	Complete	Complete
MIGS-28	Libraries used	SMRT library prep	SMRT library prep	SMRT library prep
MIGS-29	Sequencing platform	PacBio	PacBio	PacBio
MIGS-31.2	Fold coverage	94x	143x	112x
MIGS-30	Assemblers	HGAP	HGAP	HGAP
MIGS-32	Gene calling method	GeneMarkS+	GeneMarkS+	GeneMarkS+
	Locus tag	AEM38	AEM42	AEM51
	GenBank ID	CP012156	CP012157	CP012155
	GenBank date of release	March 30, 2016	March 30, 2016	March 30, 2016
	GOLD ID	Gp0126808	Gp0126809	Gp0126810
	BIOPROJECT	PRJNA290648	PRJNA290650	PRJNA290651
MIGS-13	Source material identifier	UKL13	UKL13	UKL13
	Project relevance	Environmental	Environmental	Environmental

### Growth conditions and genomic DNA preparation

One *Aphanizomenon* *flos-aquae* colony from a depth-integrated water sample from the UKL Link Dam site collected on 6 August 2013 was transferred to Bold 3 N_0_ medium [24] without NaNO_3_ (or any other form of N). This medium consisted of 0.17 mM CaCl_2_, 0.3 mM MgSO_4_, 0.43 mM K_2_HPO4, 1.29 mM KH_2_PO4, 0.43 mM NaCl, P IV trace metals, and 0.1 μM vitamin B_12_ at pH 8.0. The culture was maintained under cool white fluorescent light (20 μE m^−2^ s^−1^) with a light/dark cycle of 16 h/8 h at 24 °C. Three separate DNA extractions were performed from this culture (Table 3). A sample taken in November 2013 was collected on a 1.2 μm GF/C filter (Whatman), and DNA was extracted for Illumina sequencing using a DNA extraction kit (GeneRite DNA-EZ RWOC1). A similarly collected sample (Nov 2013) was extracted using phenol-chloroform [25] and pooled with phenol-chloroform extracted DNA from an unfiltered sample of the culture collected during March 2015 (to balance the proportion of sequencing capacity associated with cyanobacteria and heterotrophic bacteria). This pooled sample was quantified with the Q32850 Quant-iT dsDNA BR Assay Kit. Approximately eight micrograms of DNA were submitted for PacBio sequencing.

**Table 3:** DNA extraction procedures and respective sequencing technologies

Extraction	Handling	Extraction	Sample date(s)	Sequencing
1	1.2 μm GF/C filtration	GeneRiteKit (silica beads)	11/01/13	Illumina 100 bp paired-end HiSeq 2000
2	1.2 μm GF/C filtration and whole sample	Phenol-chloroform	Nov 2013 & Mar 2015	PacBio RS

### Genome sequencing and assembly

The November 2013 sample was processed using a Nextera XT kit and sequenced using the Illumina HiSeq 2000 at the Oregon State University Center for Genome Research and Biocomputing to generate 17,617,259 paired-end reads (101 bp). The pooled (11/2013 & 3/2015) sample was processed for PacBio sequencing by the Molecular Biology and Genomics Core at Washington State University. Eight SMRT cells of PacBio RS sequencing generated 348,623 reads with an average length of 7,737 bp. PacBio sequences were assembled using HGAP [26] with three different parameter sets to optimize for assembly of different genomes (Additional file 2: Table S2). Initially, only the *Bacteroidetes* genome assembled from two SMRT cells (167,289 PacBio reads), at a seed read length cutoff of 12.8 kb. The less abundant *Hyphomonadaceae* UKL13-1 and Betaproteobacterium UKL13-2 genomes required all eight SMRT cells to close (348,623 reads). While the Betaproteobacterium genome closed with a seed read-length cutoff of 13.6 kb, the *Hyphomonadaceae* genome only assembled completely when this cutoff was lowered to 6 kb, likely since it had the lowest coverage of the three genomes. A lower cutoff directs more reads towards use in assembling, thereby improving chances of completing low-coverage assemblies [27]. However, this also reduces the number of aligned reads used for error correction, which can in turn affect assembly quality. These tradeoffs should be considered before performing assemblies, but it is notable that we would not have completed the *Hyphomonadaceae* UKL13-1 genome without lowering this cutoff. The *Hyphomonadaceae*, Betaproteobacterium, and *Bacteroidetes* genomes were of finished quality (Table 4), with each having average Phred scores (ASCII base 33) of 75.9, 76.0, and 81.9, respectively. Although HGAP assembly involves chimera detection, we additionally evaluated the possibility of chimeric assembly of each genome by mapping the Illumina reads to the completed genomes using REAPR [28], which breaks incorrect assemblies by assessing the paired-read coverage distribution at each base; no chimeras were identified. We were unable to complete other genomes in the culture, including the draft-quality *A. flos-aquae* genome assembly (Table 4).

**Table 4:** Genomes identified from PacBio assemblies. PacBio read coverage calculated by mapping with BLASR

Genome	Assembly Length (bp)	No. contigs	PB read coverage	Completeness estimate	Contamination estimate
*Hyphomonadaceae* UKL13-1	3,501,508	1	94x	-	-
Betaproteobacterium UKL13-2	3,387,087	1	143x	-	-
*Bacteroidetes* bacterium UKL13-3	3,236,529	1	112x	-	-
*Aphanizomenon* flos-aquae	4,250,721	67	40x	96.67%	0.22%
Unknown *Flavobacterium*	2,347,065	96	22x	62.67%	0.25%
Unknown *Caulobacterales* bacterium	487,875	53	6x	17.15%	0.00%

Completeness and contamination estimates for incomplete genomes are from CheckM

The Illumina-sequenced culture was assembled using the IDBA-Hybrid [29] software. We binned Illumina-assembled contigs from the three completed genomes by differential coverage of reads from both PacBio and Illumina samples. That is, Illumina and PacBio reads were separately mapped to each assembly using BWA-MEM [30] and BLASR [31], respectively. Contigs were then binned using the mmgenome R package [8] (Table 5).

**Table 5:** Illumina assembly statistics for each genome. Contig number and assembly length are from extracted bins. Illumina coverage calculated by mapping with BWA-MEM. Bin coverage parameters used to bin Illumina assemblies with mmgenome

	Illumina coverage	# Illumina contigs	Bin coverage parameters	Bin assembly length (bp)	Bin assembly (% of genome)	Bin estimated completeness	Bin estimated contamination
*Hyphomonadaceae* UKL13-1	23x	122	Illumina: 15-40x PacBio: >49x	3,716,244	106.13%	98.48%	2.19%
Betaproteobacterium UKL13-2	63x	162	Illumina: 37-87x PacBio: 71-211x	3,131,899	92.47%	96.15%	1.42%
*Bacteroidetes* bacterium UKL13-3	58x	96	Illumina: 44-103x PacBio: >228x	3,009,740	92.99%	97.81%	0.55%

Assembly as % of genome is comparison of contig bin length with actual genome length. Completeness and contamination estimated with CheckM

### Genome annotation

All genomes were annotated with NCBI's PGAP [32] and PROKKA [33]. Counts of features (Genes, CDS, pseudogenes, rRNAs, tRNAs, ncRNAs, and CRISPR arrays) come from PGAP annotations. Amino acid sequences were assigned to COG categories by searching against the COG protein database [34] using RAPSEARCH [35], taking only the top hits above an E-value of 1E-30. Amino acid sequences from each genome were also annotated using the KEGG database [36] with the GhostKOALA [37] pipeline and the “genus_prokaryotes” database on September 3, 2015.

## Genome properties

**Fig. 2:**
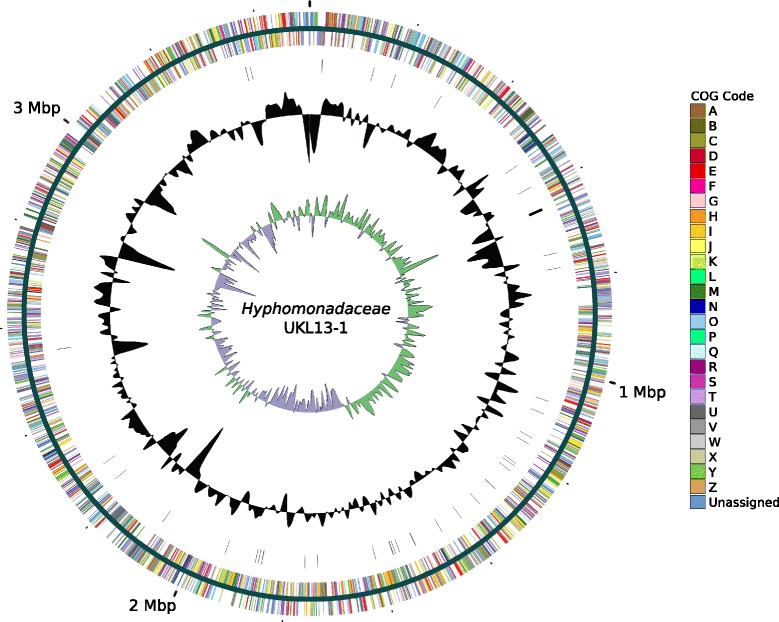
Circular map of the chromosome of *Hyphomonadaceae* UKL13-1. Circles from outermost radius to innermost: Predicted proteins encoded on the forward strand, colored by COG category; Predicted proteins encoded on the negative strand, colored by COG category; RNA genes (rRNA operons shown as thick lines); GC%, with peaks and troughs showing deviations from the average; GC skew

**Fig. 3:**
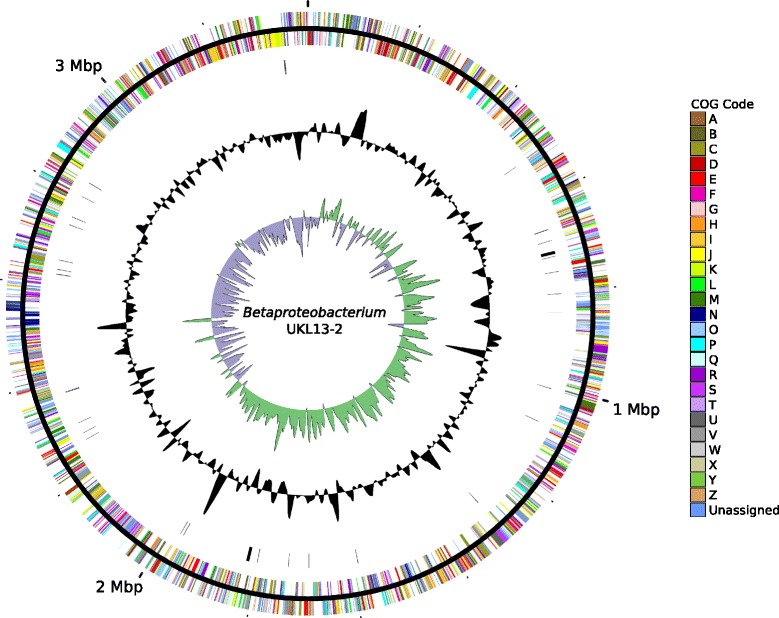
Circular map of the chromosome of Betaproteobacterium UKL13-2. See Fig. 2 legend for explanation

**Fig. 4:**
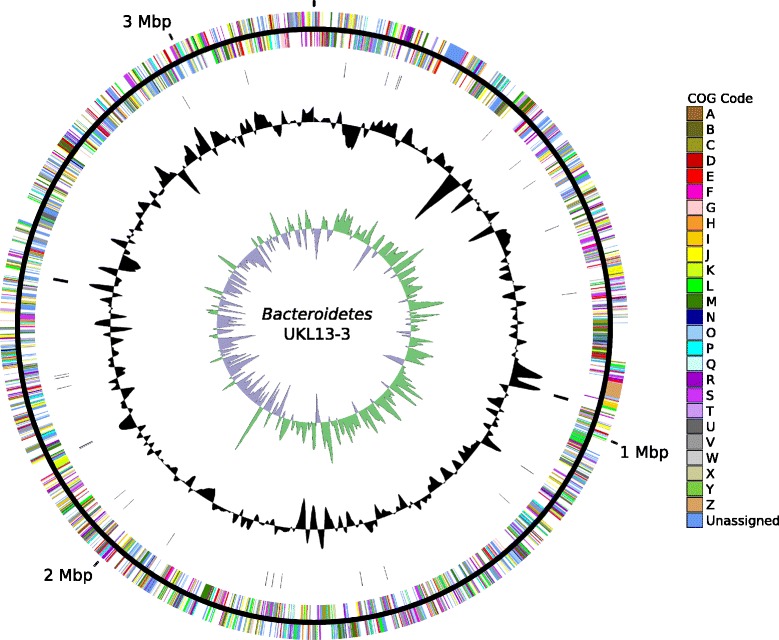
Circular map of the chromosome of *Bacteroidetes* bacterium UKL13-3. See Fig. 2 legend for explanation

Each genome assembled into one closed contig, whose properties and statistics are shown in Table 6. The *Hyphomonadaceae* UKL13-1 genome consists of a single circular chromosome 3,501,508 bp long and a GC content of 56.12%. The genome contains a total of 3255 predicted genes, including 2934 predicted protein-coding sequences, 277 pseudogenes, and 44 RNA genes (40 tRNAs, one copy of the 16S-23S-5S rRNA operon, and 1 ncRNA) (Fig. 2). The Betaproteobacterium UKL13-2 genome consists of a single circular chromosome 3,387,087 bp long and a GC content of 54.98%. The genome contains a total of 3087 predicted genes, including 2772 predicted protein-coding sequences, 265 pseudogenes, and 50 RNA genes (43 tRNAs, two copies of the 16S-23S-5S rRNA operon, and 1 ncRNA) (Fig. 3). The *Bacteroidetes* UKL13-3 genome consists of a single circular chromosome 3,236,529 bp long and a GC content of 37.33%. The genome contains a total of 2850 predicted genes, including 2598 protein-coding sequences, 211 pseudogenes, and 41 RNA genes (35 tRNAs and two copies of the 16S-23S-5S rRNA operon) (Fig. 4). The distribution of genes into COG functional categories is summarized in Table 7.

**Table 6:** Properties and statistics for each genome

Attribute	*Hyphomonadaceae* UKL13-1		*Betaproteobacterium* UKL13-2		*Bacteroidetes* UKL13-3	
	Value	% of total	Value	% of total	Value	% of total
Genome size (bp)	3,501,508	100	3,387,087	100	3,236,529	100
DNA coding (bp)	3,166,294	90.43	3,017,556	89.09	2,922,707	90.3
DNA G + C (bp)	1,964,937	56.12	1,862,116	54.98	1,208,228	37.33
DNA scaffolds	1		1		1	
Total genes	3255	100	3087	100	2850	100
Protein coding genes	2934	90.14	2772	89.8	2598	91.16
RNA genes	44	1.35	50	1.62	41	1.44
Pseudo genes	277	8.51	265	8.58	211	7.4
Genes in internal clusters	-	-	-	-	-	-
Genes with function prediction	2459	75.55	2300	74.51	1872	65.68
Genes assigned to COGs	2156	66.24	2078	67.31	1696	59.51
Genes with Pfam domains	2697	82.86	2489	80.63	2066	72.49
Genes with signal peptides	382	11.74	235	7.61	301	10.56
Genes with transmembrane helices (≥3)	310	9.52	271	8.78	255	8.95
CRISPR repeats	0		2		1

**Table 7:** Number and proportion of genes associated with COG functional categories

	*Hyphomonadaceae* UKL13-1		*Betaproteobacterium* UKL13-2		*Bacteroidetes* UKL13-3		
Code	Value	% of total^a^	Value	% of total^a^	Value	% of total^a^	COG category
J	184	4.91	187	5.07	175	5.34	Translation
A	1	0.03	1	0.03	1	0.03	RNA processing and modification
K	128	3.41	100	2.71	85	2.6	Transcription
L	109	2.91	100	2.71	126	3.85	Replication
B	2	0.05	2	0.05	1	0.03	Chromatin structure and dynamics
D	25	0.67	46	1.25	28	0.85	Cell cycle control
Y	0	0	0	0	0	0	Nuclear structure
V	69	1.84	77	2.09	74	2.26	Defense mechanisms
T	216	5.76	168	4.56	81	2.47	Signal transduction mechanisms
M	165	4.4	181	4.91	242	7.39	Cell wall /membrane/biogenesis
N	66	1.76	80	2.17	18	0.55	Cell motility
Z	0	0	18	0.49	1	0.03	Cytoskeleton
W	11	0.29	30	0.81	2	0.06	Extracellular structures
U	49	1.31	58	1.57	34	1.04	Intracellular trafficking
O	131	3.49	121	3.28	124	3.79	Posttranslational modification
C	135	3.6	187	5.07	114	3.48	Energy production and conversion
G	133	3.55	95	2.58	79	2.41	Carbohydrate transport and metabolism
E	197	5.25	224	6.08	127	3.88	Amino acid transport and metabolism
F	66	1.76	68	1.85	74	2.26	Nucleotide transport and metabolism
H	137	3.65	134	3.64	94	2.87	Coenzyme transport and metabolism
I	188	5.01	120	3.26	96	2.93	Lipid transport and metabolism
P	153	4.08	143	3.88	85	2.6	Inorganic ion transport and metabolism
Q	101	2.69	66	1.79	38	1.16	Secondary metabolites biosynthesis
R	223	5.95	213	5.78	211	6.44	General function prediction only
S	125	3.33	98	2.66	95	2.9	Function unknown
NA	1104	29.44	1083	29.39	1201	36.67	Not in COGs

^a^The total is based on the total number of predicted protein coding genes in the annotated genomes

## Insights from the genome sequence

### PacBio metagenome and comparison to Illumina metagenome

The bacterial community associated with the *Aphanizomenon* *flos-aquae* culture was subjected to metagenomic analysis with sequencing on eight PacBio SMRT cells, resulting in three completed novel bacterial genomes: *Hyphomonadaceae* UKL13-1, Betaproteobacterium UKL13-2, and *Bacteroidetes* UKL13-3 (Table 4). There were insufficient reads to close the genome of *A. flos-aquae*, although 67 contigs could be clustered to represent an estimated 97% of the genome (Table 4). The *A. flos-aquae* genome was sequenced with lower coverage than the three completed genomes, and additional sequencing would be needed for genome completion. Contigs from partial genomes of two additional bacteria were also clustered: a novel *Flavobacterium* (63% estimated genome completeness) and a novel *Brevundimonas* bacterium (17% estimated genome completeness) (Table 4), which were taxonomically placed via PhylopythiaS+. The *Flavobacterium* genome contained 16S rDNA genes with 98% similarity to *Flavobacterium aquatile* DSM 1132, but no 16S gene was identified in the *Brevundimonas* contigs. Our results indicate the presence of at least six separate bacterial taxa in this non-axenic culture.

A parallel Illumina HiSeq 2000 metagenome allowed comparison of PacBio-only and Illumina-only assemblies. When assembled with Illumina reads, the three predominant genomes separated into bins containing ~100 or more contigs. The Betaproteobacterium genome bin contained more contigs than the *Hyphomonadaceae* and *Bacteroidetes* genomes, although it was sequenced at the highest Illumina depth of the three (63x coverage vs. 23x and 58x coverage, respectively) (Table 5). There was a ~200 kb discrepancy between Illumina bin length and completed genome length for each of the three genomes. The total binned contig lengths for the *Bacteroidetes* and Betaproteobacterium were each shorter than the completed genomes, while the *Hyphomonadaceae* total binned contig length was longer (Table 5). The additional sequences in the *Hyphomonadaceae* bin were primarily contigs shorter than 10 kb that were not part of the PacBio-assembled *Hyphomonadaceae* genome. The bin quality control program CheckM [38] overestimated genome completeness or underestimated contamination when compared with the finished genome size. For example, CheckM estimated that the *Hyphomonadaceae* UKL13-1 bin contained ~2% contamination, while comparing the bin length with the completed genome length suggests ~6% contamination (Table 5). These discrepancies indicate that genome binning has a tendency to exclude important sequences or include extraneous sequences, and reveals the difficulty of assessing binned genome completeness and contamination without a reference. Incomplete binning is common for draft genomes, particularly from metagenomic assemblies [12].

We also assessed the extent to which genome repeats affected Illumina assemblies. Repeats in each genome were identified by using BLASTN to align each genome with itself, with a minimum E-value cutoff of 1E-30. Both intragenome BLASTN hits and missing Illumina coverage were then visualized with a circular genome plot (Additional file 3: Figure S1a-c). Breaks in Illumina assemblies commonly co-localized with intragenomic repeats in each genome. In particular, the Betaproteobacterium UKL13-2 genome is enriched for repeat sequences relative to the other two genomes and contains larger regions unassembled by Illumina reads, factors that possibly contributed to the greater genome fragmentation (Table 5).

**Fig. 5:**
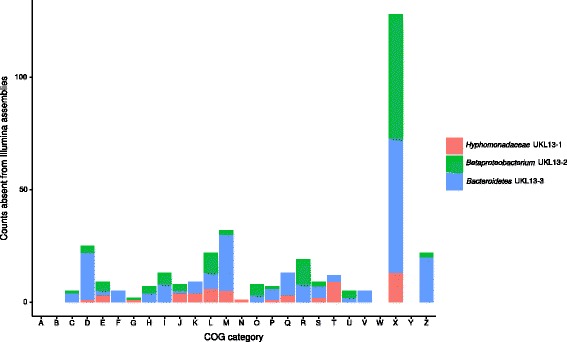
COG categories missing from Illumina assemblies determined by comparison to the closed genomes. Categories assigned with Rapsearch2. X is the mobilome COG category, while the rest of the category labels are annotated in Table 7

We then analyzed gene functions in sequences missing from Illumina bins to assess the extent to which critical gene content was missing (Fig. 5). Most annotated genes in these regions were assigned to the mobilome category (X, esp. transposases), although genes from most other COG categories were also represented. Annotations within these regions included essential genes such as tRNAs, rRNA operons, translation-associated genes, and nucleotide metabolism genes, in addition to a variety of enzymes and transporters (Table 8; Additional file 4: Tables S3, Additional file 5: Table S4 and Additional file 6: Table S5). The presence of duplicated essential genes (DNA ligase, EF-Tu) resulted in both copies being absent from the Betaproteobacterium genome (Table 8); the presence of multiple rDNA sequences commonly produces breaks in short-read assemblies [39]. In such cases, rDNA sequences confined to small contigs lose their linkage to other genes. This makes assigning 16S sequences to draft genomes difficult when multiple organisms are present in the same sample, and can make it difficult to link 16S amplicon information to binned genomes from shotgun metagenomes. Also, the functional variety of non-mobilome-associated missing genes within these assembly breaks suggests they hold informative sequences regarding physiology or lifestyle.

**Table 8:** Key genes missing from Illumina draft genome assemblies. For further details see Additional file 4: Tables S3, Additional file 5: Table S5 and Additional file 6: S5

*Hyphomonadaceae* UKL13-1	*Betaproteobacterium* UKL13-2	*Bacteroidetes* UKL13-3
tRNA-Arg	Cytochrome c4	tRNA-Asn, Ile, Ala
Inosine-5'-monophosphate dehydrogenase	Thiamine-phosphate synthase	Response regulator UvrY
16S ribosomal RNA	Rubredoxin	Ribosomal proteins S6, S18, S23, L9, L32
cytochrome b561	16S-23S-5S rRNA operons (2x)	16S-23S-5S rRNA operon
DNA polymerase III subunit alpha	tRNAs Ile (2x), Ala (2x), Asn, Ser	Riboflavin synthase
Elongation factor Tu	DNA ligase (2x)	Biotin carboxylase
50S ribosomal proteins L21, L27	Lipoprotein-releasing system proteins LolCDE	RNA polymerase-binding transcription factor DksA
	DNA polymerase III subunit alpha	Glycerol-3-phosphate dehydrogenase [NAD(P)+]
	Elongation factor Tu (2x)	

### Novel completed genomes

To functionally characterize the three novel genomes, we searched all protein-coding sequences against the COG database using Rapsearch 2.16 and a 1E-30 E-value cutoff. We then repeated this for all bacterial genomes in GenBank (collected on November 3, 2015) and compared these to our novel genomes to assess enrichment of protein-coding sequences associated with each COG functional category. These are shown as a percentage of all protein-coding sequences from each respective genome (Additional file 7: Figure S2). Our results indicate that the *Hyphomonadaceae* UKL13-1 genome contains more lipid metabolism genes than most bacteria (at 5.01% of predicted coding sequences vs. a mean of 2.96%), while the *Bacteroidetes* UKL13-3 genome contains more cell wall/envelope/membrane biogenesis genes (7.39%, vs. a mean of 4.61%).

**Fig. 6:**
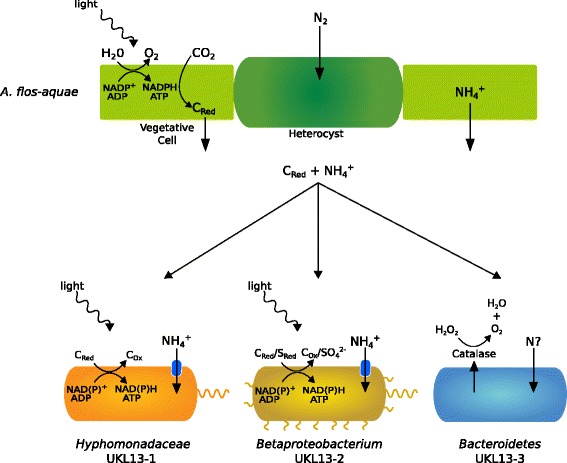
Proposed relationships between *Aphanizomenon flos-aquae* and the three novel bacteria in forming a community that is dependent on the cyanobacterium primary producer as the source of N and C, as was the case in the co-culture grown in N-free minimal medium. The *Hyphomonadaceae* and Betaproteobacterium are aerobic anoxygenic phototrophs, with likely electron donors for the production of ATP and NAD(P)H indicated. They are both motile, with flagella shown; for the Betaproteobacterium, type IV pili for twitching motility are also indicated. The *Bacteroidetes* possesses genes for gliding motility across surfaces, such as *A. flos-aquae* filaments. While ammonium importer genes are annotated for the two proteobacteria, genes for the uptake of specific forms of N were not annotated for the *Bacteroidetes*. Catalase secreted by the *Bacteroidetes* may benefit *A. flos-aquae* by reducing oxidative stress

We then searched the KEGG database to identify complete and partial pathways in each genome. Identification of additional genes was aided by using Mauve whole- or partial-genome alignments [40] to reference genomes (*Cytophaga hutchinsonii* *,* *Roseobacter denitrificans* *,* *Rubrivivax gelatinosus* *, and* *Rhodobacter capsulatus*) and between *Hyphomonadaceae* UKL13-1 and Betaproteobacterium UKL13-2. The *Hyphomonadaceae* UKL13-1 and Betaproteobacterium UKL13-2 genomes contain anoxygenic photosynthesis and reaction center genes, as well as genes for bacteriochlorophyll and carotenoid synthesis (Additional file 8: Table S6). Phylogenies of their 16S genes reveal they do not cluster near groups containing phototrophic bacteria (Fig. 1a, b). Neither genome contains RuBisCO genes, consistent with these bacteria being aerobic anoxygenic phototrophs. These are a class of heterotrophs that use phototrophy to drive ATP and NAD(P)H production, but are unable to fix net carbon through photosynthesis [41, 42]. For Betaproteobacterium UKL13-2, the presence of genes for thiosulfate or sulfite oxidation (*soxABCDXYZ*) suggests that these sulfur compounds can serve as electron donors for ATP synthesis [42], perhaps in addition to organic compounds (Fig. 6). Both *A. flos-aquae* and Betaproteobacterium UKL13-2 appear to be capable of assimilatory sulfate reduction of MgSO_4_ (provided as the only S source in the growth medium) (Additional file 8: Table S6), which is often used as the pathway for amino acid synthesis. Photolithotrophic oxidation of reduced S compounds obtained from *A. flos-aquae* by the Betaproteobacterium could be energetically advantageous. Since neither genes for oxidation of reduced sulfur or nitrogen compounds are evident in the *Hyphomonadaceae* genome, organic compounds likely serve as electron donors in this bacterium [41, 42] (Fig. 6). In contrast with the proteobacterial genomes, *Bacteroidetes* UKL 13-3 contains no autotrophy genes, consistent with the typical lifestyle of bacteria from this phylum as feeding on cellular detritus [43]. However, fewer genes were annotated from *Bacteroidetes* UKL13-3, and fewer completed KEGG pathway modules were identified than for the *Hyphomonadaceae* or Betaproteobacterium genomes (38 vs. 72 and 80, respectively). This could be due to protein-coding sequences carrying distant homology to those currently deposited in KEGG, limiting the ability to identify metabolic genes and pathways.

The *A. flos-aquae* genome was the only identified source of nitrogen fixing genes in the culture. Since the growth medium was nitrogen-deplete, all other bacteria in the community likely depend on reduced N provided by the cyanobacterium. It has been shown that *A. flos-aquae* from the Baltic Sea fixes N_2_ and releases it as NH_4_^+^, which is then taken up by surrounding heterotrophic or phototrophic bacteria [44, 45]. Both proteobacterial genomes contain the ammonia transporter gene *amtB*, which would allow uptake of NH_4_^+^ released by *A. flos-aquae* (Fig. 6)*.* No ammonia channel transport genes were annotated in the *Bacteroidetes* UKL13-3 genome. The proteobacterial genomes both contain chemotaxis and flagellar genes, and the Betaproteobacterium genome also contains type IV pilus genes for twitching motility (Additional file 8: Table S6; Fig. 6). Motility may be necessary for these organisms to stay associated with and obtain benefits from *A. flos-aquae,* similar to other host-associated bacteria [46].

We searched the novel genomes for the presence of other transporters to inform of the needs for survival and growth. Both proteobacterial genomes contain transporters for alkanesulfonate, iron(III), phosphate, and phosphonate. The *Hyphomonadaceae* genome also contains a transporter for putrescine, while the Betaproteobacterium genome contains complete transporter modules for tungstate, molybdate, glutamate/aspartate, and branched-chain amino acids. Few, and only broadly functional, transporter modules were identified in the *Bacteroidetes* genome. All three genomes appear to carry complete genetic pathways for nucleotide biosynthesis, as well as genes for synthesis of all 20 amino acids, indicating these organisms are self-sufficient in this regard. Because the *Flavobacterium* and *Brevundimonas* genomes were so incomplete (Table 4), their gene content is not reported here.

We were unable to identify any plasmids in the assemblies. The distribution of all plasmids in GenBank shows that the majority are found in *Proteobacteria* (~47%), although most of these are associated with *Gammaproteobacteria* (~63%), rather than *Alphaproteobacteria* (~22%) or *Betaproteobacteria* (~8.7%) [47]. Plasmids from *Bacteroidetes* were much rarer at ~1.6%. It may then be unsurprising that these bacteria lack plasmids.

### Freshwater bacteria associated with cyanobacterial blooms

Bacteria from the *Alphaproteobacteria*, *Betaproteobacteria*, and *Bacteroidetes* are common in freshwater systems [43], are known to be commonly associated with cyanobacterial blooms, and can directly influence the growth of cyanobacteria in culture [48]. We therefore propose that the three newly sequenced genomes represent a bacterial community that is dependent on *Aphanizomenon* *flos-aquae* (Fig. 6). Some *Alphaproteobacteria* have been identified in such cyanobacterial-associated communities [18]. For example, *Alphaproteobacteria* 16S sequences have been detected in association with the nitrogen-fixing cyanobacterium *Gloeotrichia echinulata* [49]. Interestingly, 16S rDNA from *Hyphomonadaceae* UKL13-1 shared significant identity (Additional file 1: Table S1) with one of these sequences (A0904), suggesting that bacteria related to *Hyphomonadaceae* UKL13-1 are associated with various bloom-forming cyanobacteria. However, the extent to which such co-occurrences reflect physiological interdependencies remains to be explored. *Betaproteobacteria* have been seen physically associated with cyanobacteria [18, 49]. The predicted chemotaxis and flagellar and twitching motility genes (Additional file 8: Table S6) would assist both *Hyphomonadaceae* UKL13-1 and Betaproteobacterium UKL13-2 to remain associated with *A. flos-aquae* colonies and obtain the benefits of ammonium and organic nutrients released by the cyanobacterium (Fig. 6). We have detected no genes by which these photoheterotrophic bacteria could obviously benefit *A. flos-aquae.*

Bacteria from the *Bacteroidetes* phylum are commonly identified in, and sometimes dominate, freshwater lake systems [50]. They are also frequently found in particle-associated communities and commonly degrade extracellular polysaccharide matrices that are grazed via gliding motility [51]. *Bacteroidetes* UKL13-3 possesses annotated gliding motility genes, which may indicate physical association with the originally isolated *A. flos-aquae* colony. Extracellular mucilage, as well as a range of nutrients (reduced C, N and S compounds) released by *A. flos-aquae*, may support the growth of *Bacteroidetes* UKL13-3, whose genome seems to lack many functionally annotated pathways. *Bacteroidetes* UKL13-3 has the only annotated extracellular peroxidase gene in the three genomes, which could protect against reactive oxygen species generated by photosynthesis in *A. flos-aquae* (Fig. 6), which itself lacks annotated peroxidase genes. This may indicate a mutual benefit for both bacteria, and conform to the Black Queen Hypothesis described for interactions between the unicellular cyanobacterium *Prochlorococcus* with other interacting bacteria [52]. On the other hand, large populations of *Bacteroidetes* bacteria have been observed following cyanobacterial bloom decline [53] due to subsequently favorable conditions for copiotrophs [54] and cell turnover of *A. flos-aquae* and other cells may provide organic material for *Bacteroidetes* UKL13-3 growth in co-culture, with similar benefits from lysed cells for the two *Proteobacteria*.

### Search for the novel genomes in freshwater metagenomes

We searched for the occurrence of the three novel bacteria in 62 freshwater lake metagenomes from 8 sampling sites across the United States, including Oregon, Washington state, California, Texas, and Kansas (BioProject accessions: PRJNA312985, PRJNA282166, PRJNA312830, PRJNA312986, and PRJNA294203, respectively). To do so, we mapped reads from these metagenomes to the references with BWA-MEM with default parameters (~0.067% mismatch rate) and calculated average genome coverage. Matches were found in two samples. A metagenome from Copco Reservoir, CA, on the Klamath River downstream of UKL on September 19, 2007 contained ~86x average read coverage depth of the *Hyphomonadaceae* UKL13-1 genome and ~151x coverage depth of the *Bacteroidetes* UKL13-3 genome from 398,356,734 Illumina read pairs. Additionally, a metagenome from Cranberry Lake, WA on August 11, 2014 contained the Betaproteobacterium UKL13-2 genome at ~99x average coverage depth from 13,955,857 Illumina read pairs. We also searched in 50 additional freshwater lake metagenomes in the IMG, MG-RAST, and SRA databases. The only detection found was the Betaproteobacterium UKL13-2 genome at ~19x average coverage depth in a metagenome consisting of 319,415,720 Illumina read pairs labeled “vibrio metagenome HEM-04” from a freshwater lake (BioProject accession: PRJNA64039). This initial analysis shows that the three novel bacteria are found elsewhere in freshwater habitats, although they do not appear to be ubiquitous or widely abundant.

### Taxonomic placement and naming of genomes from uncharacterized bacteria

Currently, there is a lack of guidance and standardization for assigning taxonomic nomenclature to genomic sequences lacking phenotypic information. Until now, most of these sequences have been amplicons or draft assemblies from shotgun metagenomes, in which the genomes are usually incomplete and there are likely to be some contaminating contigs. We observed both of these defects in the draft genomes assembled from the short-read Illumina sequencing that paralleled the assembly of complete genomes from the PacBio long-read sequencing (Tables 4 and 5). Critical genes—including ribosomal RNA operons—were missing from the draft genomes (Table 8), making it perhaps premature to assign new taxonomic designations to such bacteria. Our work demonstrates that as long-read sequencing depth increases, so will the likelihood of assembling complete microbial genomes from uncultured samples of high relevance to extant microbial communities. Especially for circular chromosomes, the assembly of such complete genomes would seem to carry a low risk of artifactual assembly of the genome from a mixed-DNA sample in a metagenomic study, particularly when no PCR amplifications are used in the sequencing protocol, as in the present case. It would seem beneficial to the advancement of microbial ecology to develop new guidelines by which currently unclassifiable complete genomes derived from metagenomic data can be taxonomically placed with new genus and species names as appropriate.

## Conclusions

Here, we have shown that completing multiple genome assemblies is possible from a simple microbial community using PacBio sequencing, a feat that is nearly impossible with short-read shotgun sequencing alone. There are several advantages to this approach. Completing genome assemblies from a shotgun metagenome avoids genome gaps and excludes contaminant sequences, which are significant issues with binned draft genomes. Absent sequences can contain functionally relevant information, such as gene clusters encoding secondary metabolites [55] or antibiotic resistance genes near mobile elements [56]. Here we observed that key essential genes (Table 8) were missing from each short-read assembly. Also, short-read assemblers can compress small repeats, potentially removing important functional information [57]. In addition to providing more complete genomic information, long-read sequencing of communities such as mixed cultures or environmental samples creates possibilities for new experimental designs. For example, complete genomes from novel organisms sequenced from the environment can be used as new references for culture-free resequencing efforts, such as to explore gene linkage patterns among alleles in a population. Further, long-read sequencers often detect DNA modifications, such as methylation, allowing capture of epigenetic information from environmental sequencing runs.

Although PacBio sequencing is low-throughput compared with short-read sequencers, our results suggest that the current state of this technology allows genome sequencing from communities with relatively low diversity, such as those in extreme environments [58] or when dominated by one or a few organisms [59]. Platform improvement, such as the recently released PacBio Sequel instrument, is expected to make long-read sequencing increasingly desirable for shotgun metagenomics in the future.

Here, we have sequenced three novel genomes that may be associated with *A. flos-aquae* as part of the cyanobacterial phycosphere (Fig. 6). Based on gene annotations and growth medium, both *Proteobacteria* are motile aerobic anoxygenic phototrophs that utilize fixed nitrogen and carbon provided by *A. flos-aquae.* *Bacteroidetes* UKL13-3 is a heterotroph that likely has similar nutritional requirements, and may exist in a mutual relationship with *A. flos-aquae* through provision of an extracellular peroxidase. In future work, it will be interesting to explore the possible existence and nature of dependencies between these novel bacteria and *A. flos-aquae* colonies in blooms in Upper Klamath Lake and elsewhere.

## Additional files

Additional file 1: Table S1. Taxonomic placement of each novel genome by 16S similarity, composition (PhyloPythiaS+), and multiple marker gene similarities (Phylosift). (CSV 312 bytes)
Additional file 2: Table S2. Assembly parameters for genome assemblies from PacBio reads. Minimum read length cutoff is lowest read-length used for assembly, with remaining reads used for error correction. (CSV 195 bytes)
Additional file 3: Figure S1a.
*Hyphomonadaceae* UKL13-1 genome repeats and Illumina breaks. Blue lines signify intragenomic repeats (based on BLASTN with a minimum E-value cutoff of 1E-30), and red bars mark sequences missing from Illumina assemblies. **b**. *Betaproteobacterium* UKL13-2 genome repeats and Illumina breaks. **c**. *Bacteroidetes* bacterium UKL13-3 genome repeats and Illumina breaks. (ZIP 4776 kb)
Additional file 4: Table S3. Annotated genes in *Hyphomonadaceae* UKL13-1 Illumina breaks. Genes called and annotated with PROKKA. (XLSX 13 kb)
Additional file 5: Table S4. Annotated genes in Betaproteobacterium UKL13-2 Illumina breaks. Genes called and annotated with PROKKA. (XLSX 19 kb)
Additional file 6: Table S5. Annotated genes in *Bacteroidetes* UKL13-3 Illumina breaks. Genes called and annotated with PROKKA. (XLSX 16 kb)
Additional file 7: Figure S2. Percentage of protein-coding sequences from all bacterial genomes assigned to COG categories. Novel genomes are highlighted. (PDF 73 kb)
Additional file 8: Table S6. Predicted significant genes identified in the novel genomes. Identified by PGAP annotation, PROKKA annotation, or whole-genome alignment to reference genomes with Mauve. Locus names correspond with PGAP gene calls. (XLSX 36 kb)

## Abbreviations

AAP: Aerobic anoxygenic phototroph
ATP: Adenosine triphosphate
COG: Clusters of orthologous genes
DOM: Dissolved organic matter
IS: Insertion sequence
KEGG: Kyoto encyclopedia of genes and genomes
NGS: Next-generation sequencing
PGAP: Prokaryotic genome annotation pipeline
RuBisCO: Ribulose-1,5-bisphosphate Carboxylase
SMRT: Single molecule real-time
UKL: Upper Klamath Lake
