# Supplementary figures and images for: Towards long-read metagenomics: complete assembly of three novel genomes from bacteria dependent on a diazotrophic cyanobacterium in a freshwater lake co-culture
Source: Stand Genomic Sci. 2017 Jan 19;12:9. doi: 10.1186/s40793-017-0224-8 (PMC5248499; doi:10.1186/s40793-017-0224-8)

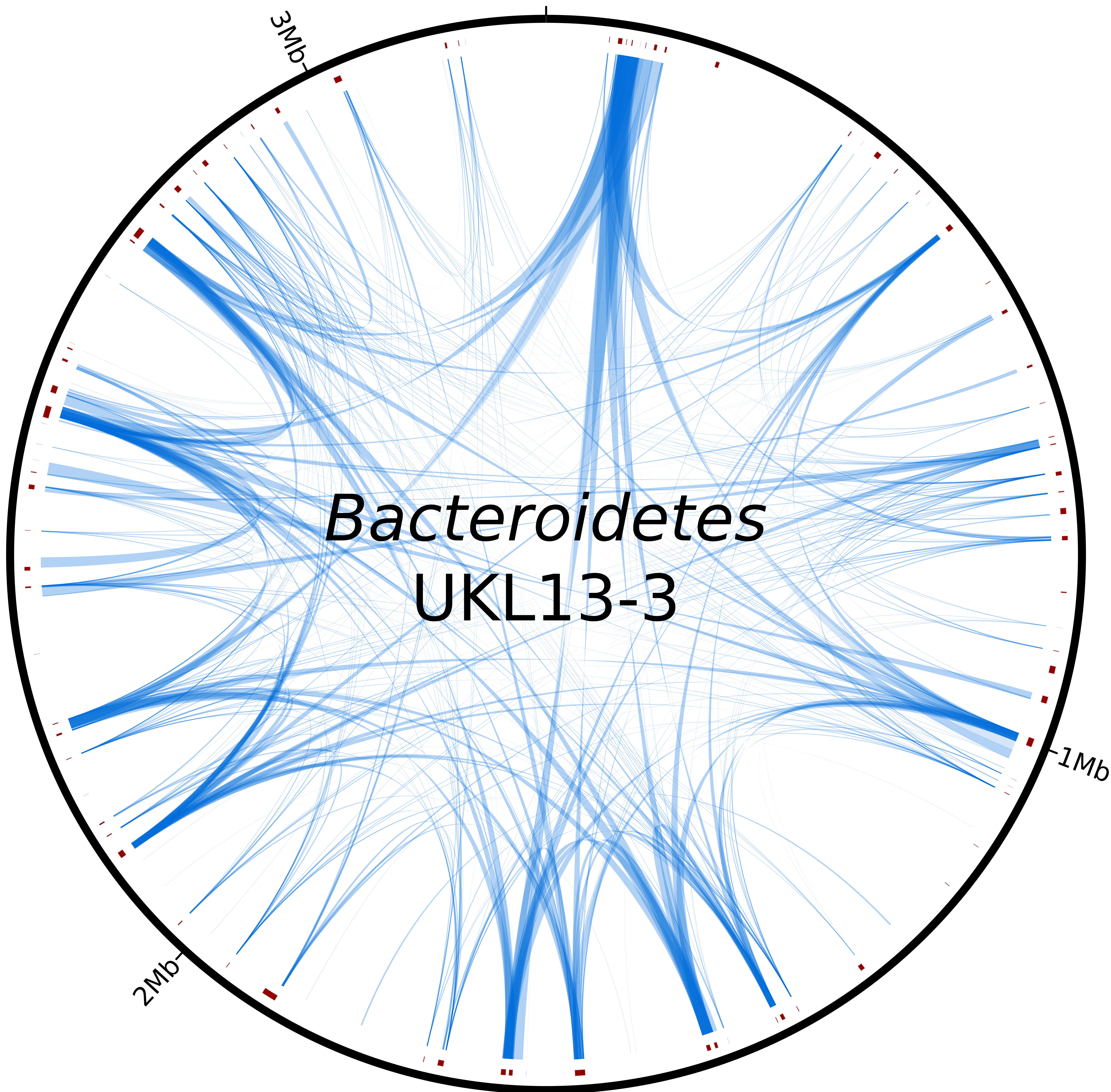

Supplement: Additional file 3: Figure S1a. — Hyphomonadaceae UKL13-1 genome repeats and Illumina breaks. Blue lines signify intragenomic repeats (based on BLASTN with a minimum E-value cutoff of 1E-30), and red bars mark sequences missing from Illumina assemblies. b. Betaproteobacterium UKL13-2 genome repeats and Illumina breaks. c. Bacteroidetes bacterium UKL13-3 genome repeats and Illumina breaks. (ZIP 4776 kb) [file 40793_2017_224_MOESM3_ESM.zip › Supp Fig S1c.pdf]

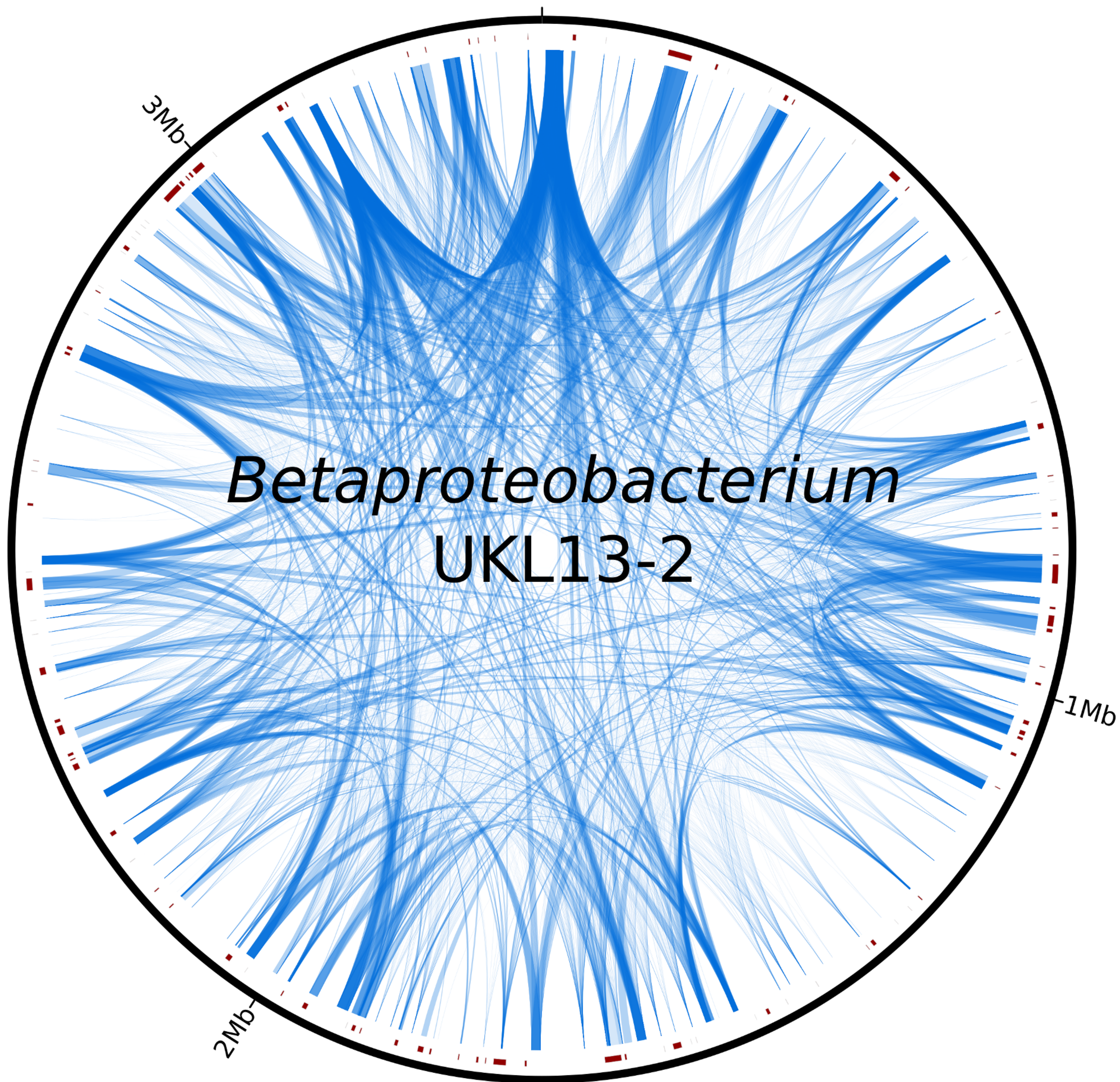

Supplement: Additional file 3: Figure S1a. — Hyphomonadaceae UKL13-1 genome repeats and Illumina breaks. Blue lines signify intragenomic repeats (based on BLASTN with a minimum E-value cutoff of 1E-30), and red bars mark sequences missing from Illumina assemblies. b. Betaproteobacterium UKL13-2 genome repeats and Illumina breaks. c. Bacteroidetes bacterium UKL13-3 genome repeats and Illumina breaks. (ZIP 4776 kb) [file 40793_2017_224_MOESM3_ESM.zip › Supp Fig S1b.pdf]

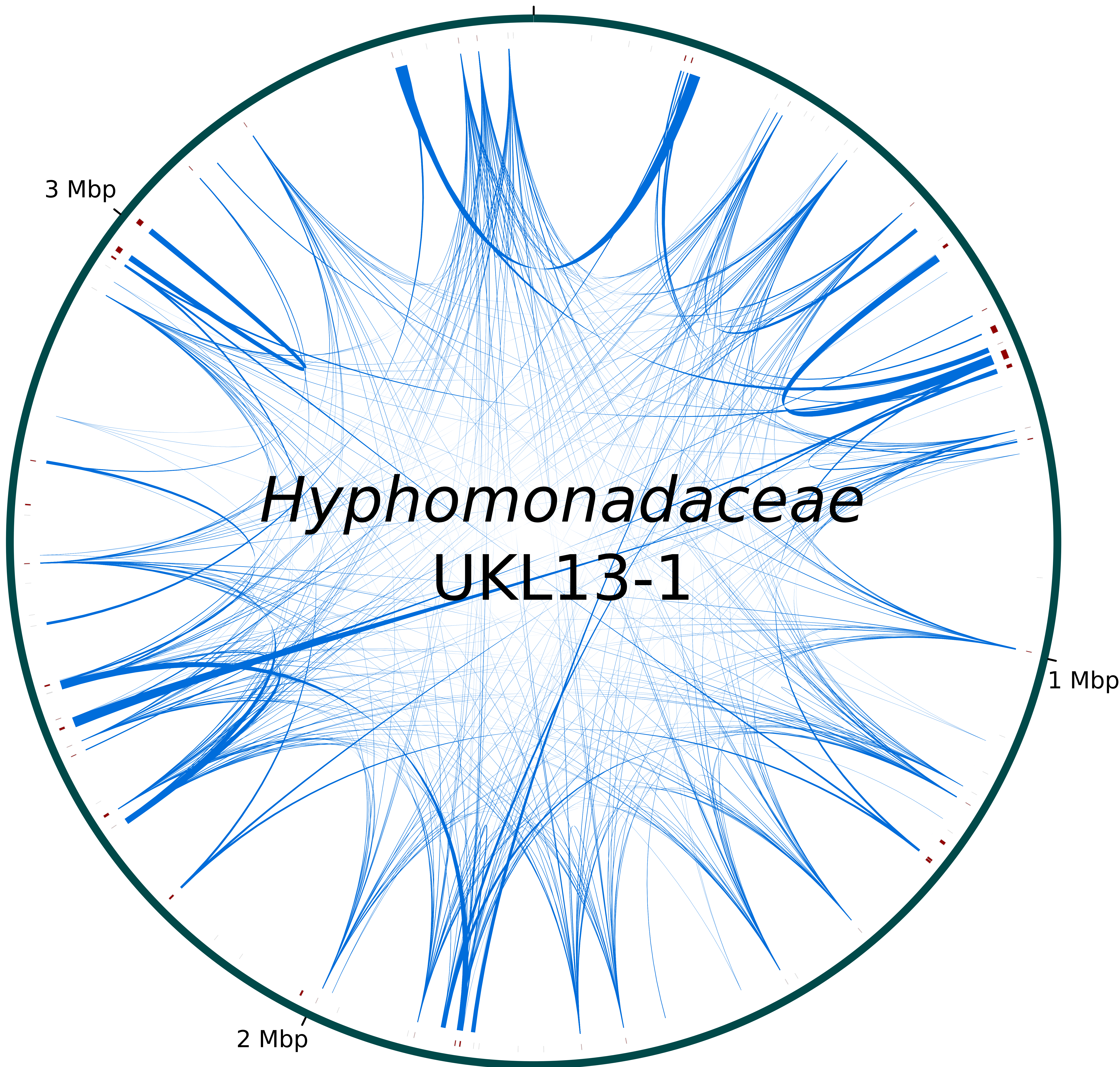

Supplement: Additional file 3: Figure S1a. — Hyphomonadaceae UKL13-1 genome repeats and Illumina breaks. Blue lines signify intragenomic repeats (based on BLASTN with a minimum E-value cutoff of 1E-30), and red bars mark sequences missing from Illumina assemblies. b. Betaproteobacterium UKL13-2 genome repeats and Illumina breaks. c. Bacteroidetes bacterium UKL13-3 genome repeats and Illumina breaks. (ZIP 4776 kb) [file 40793_2017_224_MOESM3_ESM.zip › Supp Fig S1a.pdf]

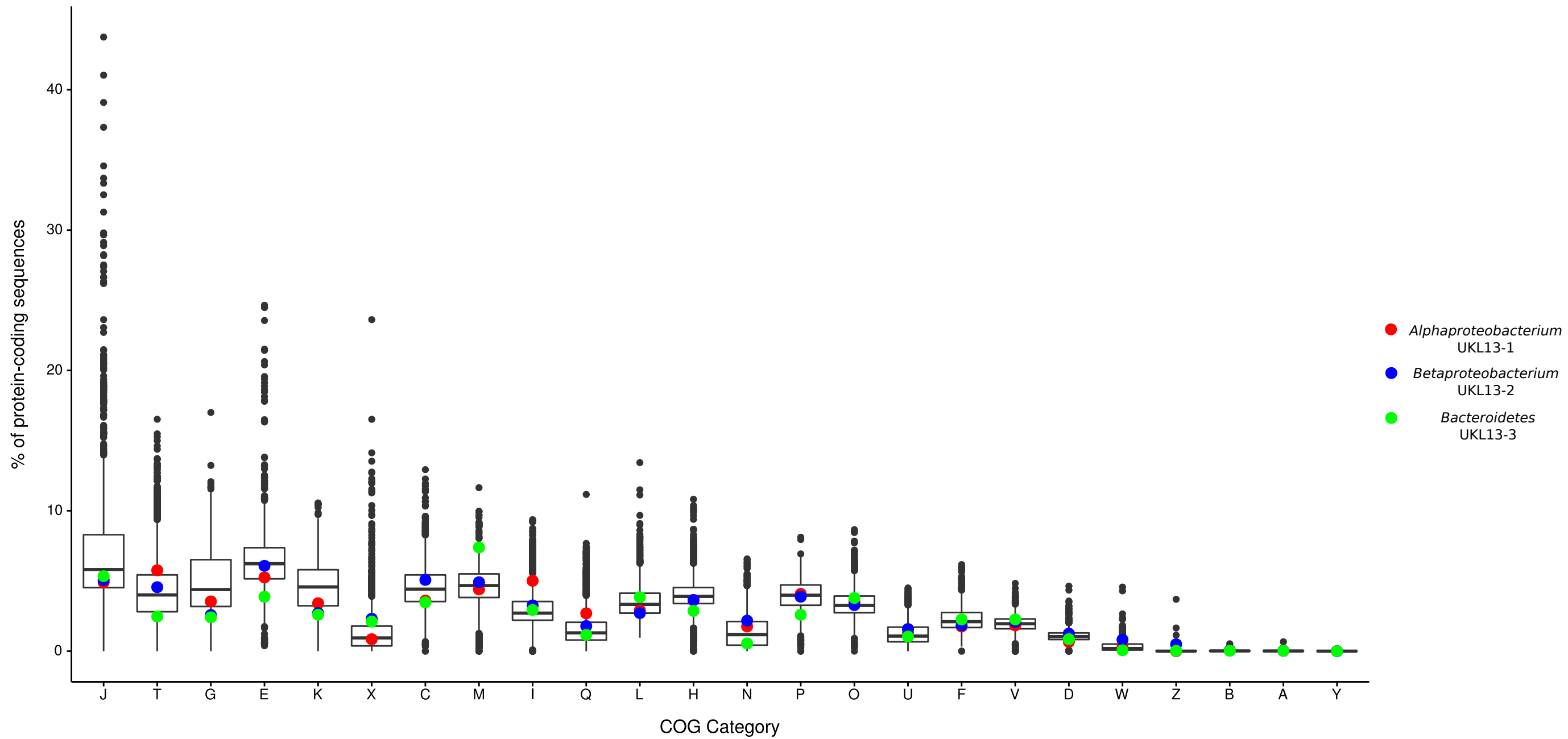

Supplement: Additional file 7: Figure S2. — Percentage of protein-coding sequences from all bacterial genomes assigned to COG categories. Novel genomes are highlighted. (PDF 73 kb) [file 40793_2017_224_MOESM7_ESM.pdf]
